# Effect of home training during the COVID-19 lockdown on physical performance and perceptual responses of team-sport athletes: a mini-review

**DOI:** 10.5114/biolsport.2022.117040

**Published:** 2022-06-27

**Authors:** Ana Carolina Paludo, Kathleen Karpinski, Shauane Emanuela Fornaciari Silva, Michal Kumstát, Zuzana Sajdlová, Zoran Milanovic

**Affiliations:** 1 Incubator of Kinanthropology Research, Faculty of Sports Studies, Masaryk University, Brno, Czech Republic; 2 Departament of Physical Education, Universidade Estadual do Centro-Oeste (UNICENTRO) Guarapuava, Brazil; 3 Department of Physical Education, Program of Post-Graduation in Physical Education UEM/UEL, State University of Londrina (UEL), Londrina, Brazil; 4 Faculty of Sport and Physical Education, University of Niš, Niš, Serbia; 5 Science and Research Centre, Institute for Kinesiology Research, Koper, Slovenia

**Keywords:** Athletes, Pandemic, Performance, Home exercise, Mental health

## Abstract

This article aims to summarize the effects of home training performed during the COVID-19 lockdown on physical performance and perceptual responses among team-sport athletes. Studies with comparison of pre-post lockdown results of physical performance and perceptual responses were considered. A search was made in PubMed and SPORTDiscus databases. The PICO criteria were used for the keywords “athlete” AND “home-based training” AND “performance” OR “mental health”, with their respective entry terms. The multistage process of selection followed the PRISMA 2020 recommendations. Of 586 records identified, 9 articles were available for the final process. Physical performance was evaluated for 8 studies with the VO_2max_ change ranging from 5.7% to -9%; an increase in the duration of sprint test ranging from 0.4% to 36%; an increase of agility duration of 12.4%; a decrease in maximal repetition load of 2.9%; and changes in countermovement jump height ranging from -4.7% to +15.4% after home training. Regarding the perceptual responses, no significant changes in wellbeing and mental index and a significant decrease in motivation and perceived effort were reported during the home training in lockdown. Based on the articles selected, home training programmes performed by athletes from team sports during the COVID-19 lockdown presented inconsistent results in physical performance, decreasing by up to 36%, and maintaining the wellbeing and mental index, but with a significant drop in training motivation and perceived effort. Caution should be taken considering the small number of articles included in the study.

## INTRODUCTION

At the beginning of 2020, a severe acute respiratory syndrome, known as coronavirus disease 2019 (COVID-19) spread rapidly throughout China to the rest of the world, causing threats to human health and lives [1]. The widespread transmission of COVID-19 led the World Health Organization (WHO) to declare COVID-19 a pandemic, and recommend public health measures such as lockdown and isolation. In sports settings, to avoid physical contact in organized training sessions, practice was forbidden at the clubs and most sports facilities. For the first time, major games and championships were suspended and/or postponed (e.g., Summer Olympics, UEFA European Football Championship) [2, 3].

In order to mitigate transmission of the virus, athletes were confined and encouraged to perform the training sessions at home. Coaches and strength-conditioning professionals faced a new challenge to plan, structure, and apply training sessions that would be appropriate for athletes to perform at home, with no or minimal utilities. In collision-based team sport, which requires physical interaction with teammates, it is also a challenge to develop or maintain game-specific contact skills (e.g., tackles, rucks, scrums) and decision-making ability [4]. Globally, this lockdown climate has caused a massive disruption in sports that has not been experienced before, and hence information regarding the effects of reduced training stimuli (detraining) in team-sport athletes during this period has been unclear [5, 6]. Recommendations about the intensity and volume of home training reinforced the importance of maintaining high loads of pre-lockdown submaximal and maximal intensity exercise to high-level athletes, in order to minimize the detraining effect [7].

Additionally, considering the pandemic situation and the changes in daily life, recommendations related to alternative activities for athletes may provide the feeling of self-control, motivation, and performing effective actions during the isolation period [7, 8]. In a global study, a survey demonstrated a desire of athletes to maintain training during lockdowns; however, the restrictions compromised aspects of training prescription (intensity, duration, and frequency), triggering a reduction in motivation in more than half of the surveyed athletes, which also possibly affected the athletes’ mental health [9].

To date, the structure of home training programmes developed by team-sport coaches and staff as well as the possible effect on athletes’ physical performance and perceptual responses has not been elucidated. Such information may help to understand what was performed during the COVID-19 lockdown and may be incorporated in similar situations, in which athletes stay at home without access to sports facilities. Also, it is important to highlight that the COVID-19 pandemic is still present, and summarizing the home training approach can provide evidence in case of a new lockdown in team sports settings. Therefore, the purpose of the present mini-review was to summarize the effect of home training performed during the COVID-19 lockdown in team-sport athletes’ physical performance and perceptual responses. A further aim was to describe the training programme used by the teams during the period that the athletes were away from the club facilities.

## MATERIALS AND METHODS

The systematic review was performed under the guidelines of the Preferred Reporting Items for Systematic Reviews and Meta-Analyses updated in 2020 [10]. Due to the main focus being the athletes’ performance, the study did not qualify for registration at PROSPERO (see the PROSPERO website for exclusion criteria: /www.crd.york.ac.uk/prospero/).

### Eligibility criteria for selecting studies

Studies were eligible for inclusion following the PICO criteria: Participants/Population (P): team sport athletes, both sexes, all age categories. Intervention/exposure (I): studies reporting the athletes’ participation in home-training programmes during the COVID-19 outbreak. Comparator/control (C): studies presenting results of variables measured before (pre) and during or after (post-) home exercises. Outcomes (O): primary outcomes included maintenance or improvement of athletes’ physical performance (maximal oxygen uptake, speed, agility, maximal repetition, countermovement jump tests) and/or perceptual measures (e.g., perceived exertion, wellbeing, motivation, mental health). Secondary outcomes included the home training description (e.g., exercises type, session duration, frequency, intensity). Studies were ineligible if the outcomes of interest were not measured or the results were not reported. Recreational athletes were not considered. Non-English language articles, reviews or guidelines, letters to the editor, conference abstracts, and dissertation theses were excluded. Only articles published between January 2020 and 30^th^ November 2021 were included.

### Search strategy and selection process

A search was performed in MEDLINE (via PubMed) and SPORTDiscus (via EBSCO*host*), during November 2021. The search terms used PICO criteria and a full search of each database was performed according to MeSH descriptors with entry terms for the PubMed database, following the descriptors with the Boolean operators “AND” and “OR” (online supplementary Table 1).

The articles from databases were imported into the Rayyan systematic review software [11] to proceed with the selection process. A multi-stage process was performed, as follows: i) one reviewer (ACP) was used to include the articles that appeared in the search strategy in each database; ii) next, the same reviewer excluded the repeated articles and then review articles and articles with non-English language; iii) two independent reviewers (KK, SEFS) screened the title and abstract and one reviewer checked all studies excluded in this phase (ACP); iv) two independent reviewers (KK, SEFS) screened the full text and one reviewer checked all studies excluded in this phase (ACP). Any disagreement between reviewers in phases iii and iv was consulted with a third reviewer (ACP).

As suggested previously [12], a prior pilot exercise with the 30 first articles was performed, demonstrating high agreement between the two reviewers (KK and SEFS with 2 disagreements). Hence, the selection was carried out with only 5 studies in disagreement, which was solved by the third reviewer (ACP).

### Data collection process

An extraction form was developed for the reviewers’ ACP and KK to extract data from each of the included studies. Extracted data included the sample characteristics (e.g., size, country, modality, sex, and age), training description (e.g., duration and components selected), and relevant outcomes (physical performance components or perceptual responses). Information that was missing from the article, an e-mail was sent to the correspondent author in order to obtain it. Afterward, the percentual variation before and after COVID-19 lockdown was calculated based on the results reported in the studies.

## RESULTS

### Included studies and characteristics

**FIG. 1:**
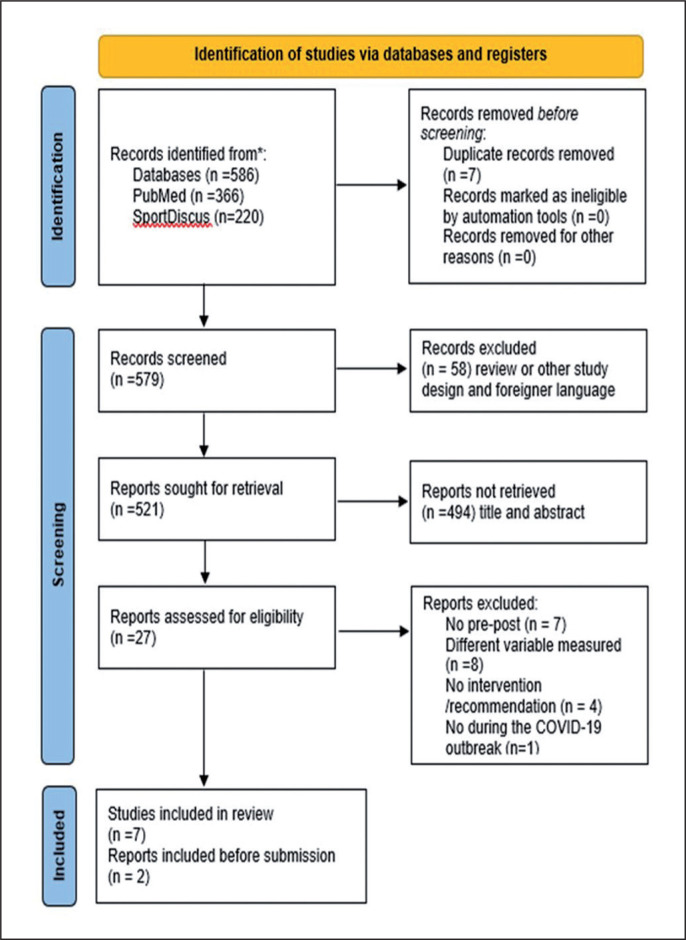
Flow chart diagram of the study selection process (PRISMA 2020).

586 records were found in the searched databases. After removing duplicates, we screened 579 records. From these records, 58 studies were excluded for presenting a review method or foreign language. Therefore, 521 were retained to screen titles and abstracts, of which 494 were excluded because they do not present a description of the home training programme and team athletes. The last phase was to take 27 articles to read the full text and after excluding the articles with no pre-post results, different variables measured, with no intervention, or not performed during the COVID-19 lockdown, 7 articles were included in the review. Later, a new search was performed during the review process. It was found 2 additional articles that fulfil the inclusion criteria were found (Figure 1).

Table 1 present the characteristics of the articles included. The studies were conducted in France [13], Germany [14], Spain [15, 21], Brazil [16], Cyprus [17], Norway [18], Hungary [19] and Italy [20]. Five articles evaluated football players [13, 17–20], one article evaluated basketball [16], two articles handball [14, 15], and one article futsal athletes [21]. Only two articles included female athletes [16, 18].

**TABLE 1:** Characteristics of the included studies and relevant outcomes.

Sample characteristic			Training program				Outcomes							
Study	Sport (country)	Gender, Age (yrs), sample size	Pre-Post (month)	Training duration (weeks)	Frequency (weekly; duration)	VO_2max_ (mL/min/kg)	sprint (sec)	agility (sec)	RM (kg)	CMJ (cm)	RPE (a.u)	WB (score)	motivation (score)	mental index
Dauty et al. [13]	Soccer (France)	M	March/May	8	4x; 45 min	9% ↓*						↔		↔
		13–14												
		N = 25												

Fikenzer et al. [14]	Handball (Germany)	M	July 2019/May	8	3x	5.7%↑								
		N = 10												

Font et al. [15]	Handball (Spain)	M	January / May	9	5x; 46–55 min					↔				
		27.9 to 29.5 ± 7.0												
		N = 11												

Moscaleski et al. [16]	Basketball (Brazil)	F	February/July	22	5x; 75–98 min						↓*	↔	↓*	
		25.7 ±7.0												
		N = 5												

Parpa & Nichaelides [17]	Soccer (Cyprus)	M	July 2019/May^#^	7	7x	3.6%↑*				15.4.%↑*				
		27.68 ±5.99												
		N = 19												

Pedersen et al. [18]	Footbal (Norway)	F	March/June	12	233 ± 47 min		0.4%↑		2.9%↓	4.6%↓				
		18.8 ±1.9												
		N = 9												

Pucsok et al. [19]	Soccer (Hungary)	M	February/June	13	3 x; 60 min			12.4 ↑		4.1%↑*				
		16.7 ±0.6												
		N = 11												

Rampinini et al. [20]	Soccer (Italy)	M	February/May	13	8 sessions per week					↔				
		25.4 ± 5.0												
		N = 50												

Spyrou et al. [21]	Futsal (Spain)	M	March/May	10	2–3 x		36%↑*			4.7%↓				
		26.7 ±3.1												
		N = 10												

Note: N – sample size; M – male; F – female; ↑* – significant increase; ↑ – increase; ↓* – significant decrease; ↓ – decrease; ↔ – no significant difference. RM = maximal repetition; CMJ = countermovement jump test; WB = wellbeing; A.U = arbitrary unity.

# = Information provided by the authors.

Outcomes of the included articles were VO_2max_ [13, 14, 17], sprint [18, 21], agility [19], maximal repetition (RM) [18], and countermovement jump test (CMJ) [15, 17–19, 21] regarding the physical performance and rating of perceptual effort of the session (s-RPE) [16], wellbeing [13, 16], motivation [16], and mental index [13] regarding the perceptual measures.

### Physical performance

The physical performance was evaluated in 8 out of 9 articles, presenting different responses amongst them. The VO_2max_ decreased significantly in soccer athletes (9%) [13], but showed an increase of 3.6% in soccer athletes [17] and 5.7% in handball athletes, although not significantly [14]. Time in sprint tests decreased significantly in futsal athletes [21] but not in football athletes [18]. Impairments of 12.1% were found in the agility of soccer athletes [19] and 2.9% in maximal repetition [18]. The CMJ test was performed in six studies in which two studies demonstrated a significant increase in soccer athletes’ jump height from 4.1% in Pucsok et al. study [19] to 15.4.% in Parpa and Nichaelides study [17], and one study reported a significant decrease of 4.7% in futsal athletes [21], and three studies, also in soccer athletes, showed no pre- to post-lockdown difference [15, 18, 20].

### Perceptual measures

Unlike the physical performance assessed by the articles on pre-post COVID-19 lockdown, the perceptual measures were monitored weekly in the two studies selected [13, 16]. The studies reported that the wellbeing did not change significantly during the training programme either for male soccer athletes [13] or female basketball athletes [16]. Furthermore, the soccer athletes also did not change their mental responses. Nonetheless, the basketball athletes reported a significant drop in motivation to train during the lockdown, together with a decrease in perceived effort regarding the training intensity.

### Training programme characteristics

The studies presented the first measure of physical performance before the COVID-19 lockdown, in February 2020 [19, 20] and March [13, 18, 21] (Table 1), reporting that the team was in the preparatory season [18] and even in the transition/rest period [20] or already the in-season period [14, 16, 21]. Two studies were used as pre-lockdown measures, outcomes from a previous season [15, 17]. The frequency of the training performed at home varies from 2–3 to 7 times per week. The exercises during the home training presented the following characteristics: strength exercises performed with the athletes’ body weight [20, 21]; resistance exercises involved running recommendations outside [14, 21] and in-home stationary equipment devices such as treadmill or bike [15, 20]; plyometrics, sprints and ball control were used as complementary exercises during the sessions [16, 18].

Regarding the prescription of the home training programme, studies vary from individual training plans, with individual workouts [14, 15, 17, 18, 19, 21], demonstrations of the exercises using videos [13], to online sessions together with a staff-team member [16]. Contact via online platforms (e.g., WhatsApp) was used by athletes to send information such as perceived session intensity, motivation [16], wellbeing [13, 16], and mental index [13] (Table 2).

## DISCUSSION

In the current review, we summarized the effect of home training performed during the COVID-19 lockdown on physical performance and perceptual responses among team-sport athletes, and as major findings, the physical performance decreased in most studies, with a negative effect of up to 36%. Additionally, athletes presented no changes in wellbeing, reported in two studies, and a significant drop in their perceived intensity and motivation to train, but no changes in mental-index during the programme were described in one study.

### Physical performance and home-training characteristics

The VO_2max_ showed a significant decrease of 9% in young soccer athletes [13] and an increase of 3.6% in adult soccer [17] and 5.7% in handball athletes [14]. This difference could be explained by the distribution of the physical components performed during the home training. The young soccer athletes performed only 2 sessions a week dedicated to the aerobic system [13]. Continuous and high-intensity intermittent training (HIIT) running exercises during this period were planned to be around 80% of the maximum heart rate as well as the reduced duration in this session compared to usual training (45 to 75 min prior to and 45 min during lockdown) [13]. On the other hand, the handball athletes performed 3 sessions per week of endurance exercises, including continuous (70–80% HRmax) and intermittent (HIIT) running, both short and long (85–90% HRmax) [14]. Also, the 3.6% the increase in VO_2max_ in adult soccer athletes can be due to home training workload volume, in which they were performed 4 times per week, with different exercises in each training session: sprints (Tuesday), seep intervals (Thursday), tempo intervals (Saturday) and continues running for 45 min (Sunday). Thus, at least 3 sessions per week can be beneficial to improve the maximal oxygen consumption in athletes during home training programmes.

**TABLE 2:** Characteristics of the training during the COVID-19 lockdown.

Components (weekly performed)							home training characteristics
Study	strength	endurance (aerobic)	Stretching/mobility	plyometric	sprint/acceleration	sport-specific (ball-control)	
Dauty et al. [13]	2 x	2 x	1 x				Multimodal program in order to stimulate all physical abilities of the players, considering the development of the young athletes in this age. Monday: Cardio-training (45 min); Tuesday: Lower leg strengthening (45 min); Wednesday: Upper legs strengthening (45 min); Thursday: rest or stretching; Friday: High-intensity interval training (45 min); Saturday: Rest or sophrology, or juggling Sunday: Rest. The demonstration of the exercises was carried out using videos addressed to the players via the web.
Fikenzer et al. [14]	2 x	3 x					The individual training plan consisted of three training days (days 1–3) with specific contents and was designed in such a way that there were always 2 training days in a row and then the following day was free (e.g., Monday: day 1, Tuesday: day 2, Wednesday: free, Thursday: day 3, Friday: day 1, the weekend was always free, Monday: day 2, Tuesday: day 3, etc.). Day 1 included stabilization training and endurance I exercise; day 2 included strength training legs and endurance II exercises; day 3 included strength training upper body and endurance III exercises.
Font et al. [15]	3 x	2 x	5 x				Structural training programs were sent each week to perform at home, five days (Monday to Friday), with a break during the weekend. Weeks 1–4: Endurance = Circuit training/Strength-based HIIT (RPE 5–8), 45.8 ± 12.3 min duration. Strength = 3 × 30 seg work and 30 seg rest / 3 × 12 rep. with self-selected recovery (1–2 min range), 50.1 ± 10.6 min duration; Weeks 5–9: Endurance = Aerobic Fitness/stationary bike (RPE = 5–7) or outdoor continuous running (RPE = 4–6), 53.3 ± 10.9 min duration. Strength = Hypertrophy-oriented program, super-sets with low specificity level preceded slightly more specific exercises. 3 × ˜3–4 exercises super-sets of 10 rep of each exercise, with self-selected recovery (1–2 min range), RPE = ˜7, 54.9 ± 12.8 min duration.
Moscaleski et al. [16]	5 x	2–3 x		5 x	2 x	2–3 x	The basketball athletes trained 6-weeks in usual training (phase 1), and the home training started after the first match of the season. Phase 2 = Mondays, Wednesdays, and Fridays: warm-up with stationary exercises, conditioning training sessions (30 min) on the treadmill, stationary bicycle, or jogging; and strength training sessions for upper body (75 min). Tuesdays and Thursdays: agility and balance exercises performed in a circuit, adapted for small spaces; and strength training sessions for upper body (75 min). After five weeks (phase 3) the conditioning sessions focused on technical performance (e.g., general basketball skills), and were added plyometric and cognitive-technical exercises, video analysis, and psychological sessions.
Parpa & Nichaelides [17]	3 x	4 x	3 x				The training protocol was identical each week and included: Strength training session on Monday (6 exercises, 4–5 sets, 4–8 rep; exercise elevated pushups: 4–5 sets, 12–15 rep), Wednesday (6 exercises, 5 sets, 4–8 rep; exercise elevated pushups: 4–5 sets, 12–15 rep), and Friday (circuit with 9 exercises 30s:20s). Cardiovascular sessions were recommended for Tuesday (sprints), Thursday (speed intervals), Saturday (tempo intervals), and Sunday (continues running for 45 min).
Pedersen et al. [18]	40 ± 39 (min)	45 ± 39 (min)			26 ± 19 (min)	95 ± 106 (min)	The team was in the pre-season, and the lockdown was introduced 4 weeks prior to the planned start of the competitive season. The training program during the lockdown included weekly football training (95 ± 106 min), strength training (40 ± 39 min), speed and jump training (26 ± 19 min), endurance training (45 ± 39 min). It was performed as individual (2.0 ± 2.0 sessions) and group/team (1.8 ± 1.7 sessions) training.
Pucsok et al. [19]	1 x	1 x	3 x	1 x	1 x	1 x	It was performed three training sessions, with 60 min of duration each. Training session one: improve endurance with regular outdoor running for 12 min, repeated three times, 2 min of recovery time, set intensities at seven on the RPE scale. Training session two: strength-endurance training was performed (both upper and lower limbs), in which athletes performed twelve exercises, repeated three times with their body weight. incorporated a load/rest ratio of 25–35 s, 30–30 s, and 35–25 s, in three periods. Athletes performed 2.5 min of passive rest at the end of each set. Training session three: multi-directional movements and jumps. Training session “A” was performed in uneven weeks, including four multidirectional runs and micro-movements. In contrast, session “B” was performed in even weeks and included four plyometric jumps to improve reactive strength. We designed the exercises for the participants to perform even in a relatively small 5 × 5 m^2^ area (room).
Rampinini et al. [20]	2–3	4–5					Training week activities description: 4 or 5 aerobic sessions performed at medium to high intensity on in-home stationary equipment devices (treadmill or bike) +2 or 3 strength training sessions using body weight and small weights.
Spyrou et al. [21]	2–3 x			2–3 x	2–3 x		The team was in the in-season period before the lockdown. Home-training followed a semi-structured maintenance program, comprising exercises using only the body mass as resistance (e.g., vertical and horizontal jumps, half and full squats, lunges, push-ups). Athletes were instructed to perform these exercises 2–3 times per week, with 2 or 3 sets of 6–8 (jumps) and 10–12 (squats and lunges) repetitions.

**Note:** HIIT = High-Intensity Interval Training; RPE = Rating of Perceived Exertion.

The performance on the sprint test decreased for both female football athletes [18] and male futsal athletes [21] after lockdown. A major negative impact was reported by futsal athletes (decrease up to 36%), which the authors described as large and significant (ES = 1.31). The decrease can be explained by the structure of the training programme, in which no sprint training was recommended during the home training. The authors reported that before the lockdown, the futsal athletes performed 5 to 8 repetitions of 10 m sprint at high intensity [21]. The female football athletes demonstrated an impairment in the time on the sprint test of only 0.4%, which might not be significant considering the training condition. During the home programme, the coaches incorporate speed exercises 2–3 times per week, and this can account for the small reduction in sprint performance [18].

The agility and maximal strength both were impaired after home training. The agility was evaluated only in one study, in young soccer athletes using the SpeedCourt device, and a decrease of 12.1% in athletes’ performance was found. The home training was performed by the soccer athletes 3 days per week, and on only one day the agility and acceleration drills were performed together with multidirectional exercises and jumps [19]. The maximal strength, which consisted of one maximal repetition of the partial back squat test, was evaluated in female football athletes. The high load chosen by the athletes to perform the exercise decreased by 2.9%. During the home training, the weekly volume of strength training increased by 43% compared to conventional training. It seems that even with inadequate facilities to perform strength exercises, the increase of training volume attenuated a significant drop in maximal repetition, based on the back squat test [18].

Finally, the countermovement jump (CMJ) test performed pre-post COVID-19 lockdown presented a range of results between the articles. A significant improvement in jump height of 15.4% and 4.1% was demonstrated in soccer athletes, from Parpa and Nichaelides study [17] and Pucsok et al. study [19] respectively. The athletes from Pucksok et al. study performed one training session per week with specific jump exercises, which could be the reason for the increased performance on the jump test [19]. No changes in CMJ outcomes were found in Rampinini et al [20] and Font et al. [15] studies. Male soccer players from the Rampinini et al. study [20] performed training focused on aerobic (4–5 sessions) and strength training (2–3) using bodyweight, without jump exercises, and the CMJ results did not show a significant pre-post lockdown change. Similarly, the handball athletes from Font et al. study [15] did not differ significantly in the values of jump height, even with a reduction of 40% in workload volume during the home training period *versus* normal training. Also, the athletes performed jump exercises only during the 4 weeks (from a total of 9 weeks), during the endurance training. There was a similar decrease, of 4.6 and 4.7% in jump height, in female athletes from the Pedersen et al. [18] study and futsal athletes from the Spyrou et al. [21] study. In both teams, male futsal athletes [21] and female football athletes [18] practised exercises such as vertical and horizontal jumps, at moderate intensity, during their home training, and such a training prescription would help attenuate the decrease in CMJ performance.

Taking the results together, it is possible to note that physical performance components that had no significant changes, or small decreases, were related to corresponding exercises performed in the home training (for example, jumps and sprints), with no reduction frequency (time) and intensity, compared before the training lockdown. The significant negative changes, such as in aerobic capacity and sprinting, could be due to reduced training stimuli during the home training. The information about the physical performance reinforces the concern described in the Bisciotti et al [6] review, in which the authors suggested that caution should be taken when athletes return to normality, after a lockdown period or home training. The short time return to sports routine can face problems such as loss of performance and the increase in injury risk during the regular season [6].

### Perceptual responses

In periods of lockdown, monitoring athletes’ motivation, wellbeing and mental health during periods of lockdown should be considered as part of a home programme. In the current review, only two studies reported perceptual measures such as wellbeing, mental index, motivation to train, and perceived effort during the home training. Regarding wellbeing and the mental index, they did not change throughout the weeks of exercises at home in female basketball athletes [16] or in young male soccer players [13]. The authors from both studies speculated that athletes maintain the wellbeing and mental index due to the online communication between the athletes and team staff. The virtual relationship could contribute to the maintenance of good psychological responses.

Despite the online contact of team staff during the training sessions, it did not prevent a drop in motivation to train for basketball athletes. The study by Moscaleski et al. [16] was the only one that monitored the athletes’ motivation to train during each training session of the home programme and showed that when the training session was transferred to home, the athletes’ motivation dropped significantly. Similar articles, with a cross-sectional approach, also demonstrated that most athletes experienced decreased motivation to train compared to pre-lockdown [22, 23]. To motivate the athletes, Moscaleski et al. [16] described the application of extra activities such as virtual challenges, match analysis was organized and also sessions with a psychologist were proposed but did not help. The authors explored the hypothesis that the absence of training in team facilities and absence of personal contact with the teammates could negatively affect the motivation to train. Similar results were reported in a global survey with athletes from different sport modalities and levels, in which training modifications can be a factor related to reducing motivation [9]. Moreover, motivation is also related to a reward, and in sports settings, athletes train focused on performing the best in competition. The authors suggested that as the lockdown lasts, with no certainty to end, the lack of real goals (competition), together with the restriction of the social training environment, could affect systems related to motivation [16].

The perception of the training intensity over each home session was reported also in the Moscaleski et al. [16] study and demonstrated a significant decrease in rating perceived effort of the training session (s-RPE) during the home training. The reduction in perception of training intensity was excepted due to the difficulties for an appropriate prescription and stimulus during the period of home training. After 5 weeks of home training, training duration was increased as a means to induce a sufficient stimulus along with additional exercises (e.g., postural stability, speed, and technical/specific activities), which also increase the overall training volume. Still, a change in perceptual training intensity measure during the home training, relative to pre-lockdown was not observed [16]. These results reinforce the limitation on home training during the lockdown, especially in training structure, in increasing the level of the training sessions.

It is important to highlight that during the COVID-19 situation, another strategy was used to mitigate the effects of lockdown in athletes, called “bubble” training camps. The “bubble” is a quarantine-style camp with coaches, athletes, and support staff, strategically isolated from the outside world to follow a normal training practice [24]. This strategy can provide better dietary habits, training routines, and well-being compared to home lockdown, in elite and world-class athletes [25]. Considering team sports, it is worth high-lighting that the “bubble” may not be affordable due to the large number of people involved. Therefore, the current review demonstrated that the application of home training can be helpful. Based on the included studies, the impact of home training on athletes’ physical performance resulted in a decrease of up to 36%, so if the lockdown lasts longer or a similar situation happens again, improvement in training structure together with monitoring perceptual responses such as training intensity and motivation can be an option to avoid a significant drop in physical performance.

### Limitations of the study

Although this mini systematic review presents for the first time the effect of home training on team-sport athletes, there are limitations to bear in mind. The lack of studies investigating the current topic and the different physical performance components and perceptual measures evaluated amongst the studies could be the major limitation on the generalization of the results. Variables for which the results came from only one study should be taken into consideration.

## CONCLUSIONS

Considering the unexpected and unprecedented period in the settings of sport, the studies demonstrated that the team sports athletes started their training at home soon after the first COVID-19 lockdown. The training programme performed presented inconsistent results in physical performance (VO_2max_, sprint, agility, maximal strength, countermovement jump), decreasing by up to 36%. Moreover, training by correspondence (virtually between team staff and athletes) during the lockdown situation was practical and positively affected the wellbeing and mental index of athletes. Nonetheless, one article reported that home training decreased the athletes’ motivation to train and perception effort in each session. Improvement in load (volume) and intensity should be considered during home training, in order to promote significant physical changes in athletes, together with strategies to improve their motivation to train.

## Conflict of Interest Statement

The authors have no conflict of interest relevant to this article.

## Funding/Support Statement

No financial or material support of any kind was received for the work described in this article.

The author ACP is supported by Operational Programme Research, Development and Education – Project “Postdoc2MUNI” (No.CZ.02.2.69/0.0/0.0/18_053/0016952).
